# Awake Craniotomy in Conscious Sedation: The Role of A2 Agonists

**DOI:** 10.3390/brainsci14020147

**Published:** 2024-01-30

**Authors:** Antonio Izzi, Giuseppe Mincolelli, Grazia D’Onofrio, Vincenzo Marchello, Aldo Manuali, Nadia Icolaro, Lucia Mirabella, Anna Riefolo, Barbara Mazzotta, Alessio Barile, Leonardo Pio Gorgoglione, Alfredo Del Gaudio

**Affiliations:** 1 UOC of Anesthesia and Resuscitation II, Fondazione IRCCS Casa Sollievo della Sofferenza, San Giovanni Rotondo, 71013 Foggia, Italy; antonioizzi1201@gmail.com (A.I.); mincolellig@gmail.com (G.M.); vincenzomarchello@libero.it (V.M.); a.manuali@operapadrepio.it (A.M.); barbara.mazzotta.bm@gmail.com (B.M.); a.barile88@gmail.com (A.B.); freddydelgaudio@libero.it (A.D.G.); 2 Health Department, Clinical Psychology Service, Fondazione IRCCS Casa Sollievo della Sofferenza, San Giovanni Rotondo, 71013 Foggia, Italy; 3 Complex Unit of Neurosurgery, Fondazione IRCCS Casa Sollievo della Sofferenza, San Giovanni Rotondo, 71013 Foggia, Italy; nadia.icolaro@gmail.com (N.I.); l.gorgoglione@operapadrepio.it (L.P.G.); 4 Department of Medical and Surgical Science, University of Foggia, 71100 Foggia, Italy; lucia.mirabella@unifg.it; 5 Anesthesia and Intensive Care Unit, Azienda Ospedaliero-Universitaria Consorziale Policlinico di Bari, 70124 Bari, Italy; 20annariefolo17@gmail.com

**Keywords:** awake craniotomy, monitored anesthesia care, α2-agonists, Dexmedetomidine, clonidine, remifrentanil

## Abstract

Background: In Awake Craniotomy (AC), α2-agonists and remifentanil (clonidine and dexmedetomidine) are used in the preoperative phase and throughout the procedure to combine monitored anesthesia care and local anesthesia. The study aims were to specify the key role of α2-agonists administered and to evaluate complication presence/absence in anesthesiologic management. Methods: 42 patients undergoing AC in 3 different centers in the south of Italy (Foggia, San Giovanni Rotondo, and Bari) were recruited. Our protocol involves analgo-sedation by administering Dexmedetomidine and Remifentanil in continuous intravenous infusion, allowing the patient to be sedated and in comfort but contactable and spontaneously breathing. During pre-surgery, the patient is premedicated with intramuscular clonidine (2 µg/kg). In the operating setting, Dexmedetomidine in infusion and Remifentanil in Target Controlled Infusion for effect are started. At the end of the surgical procedure, the infusion of drugs was suspended. Results: There were no intraoperative side effects. The mean duration of interventions was 240 ± 62 min. The average quantity of Remifentanil and Dexmedetomidine infused during interventions were 4.2 ± 1.3 mg and 1.0 ± 0.3 mg, respectively. No significant side effects were described in the post-operative phase. A total of 86% of patients and 93% of surgeons were totally satisfied. Conclusions: Synergy between opioid drugs and α2 agonists plays a fundamental role in ensuring procedure success.

## 1. Introduction

Over the last years, the number of interventions on brain tumors in the Broca area has increased as a consequence of the development of the Awake Craniotomy (AC), a procedure that allows mapping of language areas, thus limiting the damages related to surgical resection. AC, through the improvement of anesthesiological management, has now become the “gold standard” technique for neurosurgical tissue resections in the Broca area [1]. The use of drugs that act by modulating consciousness and pain ensures patient comfort during surgery. The management strategy of a patient who has to undergo an operation of such invasiveness is certainly complex since it requires both immobilizing the patient and obtaining his/her collaboration [2]. In order to obtain an immobilized and cooperative patient, it is necessary:

(1) Correctly choose the drugs for analgo-sedation, taking into account the intrinsic pharmacokinetic (PK) and pharmacodynamics (PD) characteristics of each drug [3].
(2) Carry out correct loco-regional anesthesia such as the Scalp Block [4].

During the monitored anesthesia care (MAC) technique, the dosage and pharmacological infusion are tailored by exploiting the synergy between the α2-agonists and remifentanil thanks to their pharmaceutical characteristics [3].

The two common α2-agonists used clinically are clonidine and dexmedetomidine. Although dexmedetomidine has been used successfully in regional analgesia and anesthesia [5], there are only very few studies evaluating the peripheral analgesic effects of dexmedetomidine.

Our protocol consists of a multifactorial anesthesiological technique, which includes the use of locoregional anesthesia (AL), Remifentanil in continuous intravenous infusion in Total Intravenous Anesthesia-Target Controlled Infusion (TIVA-TCI), and the use of a2-agonist drugs (clonidine and dexmedetomidine), both in the preoperative phase and throughout the procedure [6]. These drugs are used in an attempt to combine MAC and local anesthesia. The fundamental concept of this treatment is to specify the key role of the α2-agonists administered, considered the fulcrum of such anesthesiological management [7,8].

It is considered as a primary end-point: (1) the failure rate of conscious sedation through the possible need to switch to general anesthesia before mapping the affected areas and surgical resection of the cerebral tumor.

The following items were considered as secondary end points: (1) desaturation episodes; (2) finding of significant hemodynamic instability through the detection of alteration of the parameters observed on the monitor, in particular heart rate (HR), average blood pressure (ABP) and value derived from the pulse pressure contour or the Stroke Volume all through a possible variation of 20% of the basal values; (3) the presence of symptoms such as nausea and vomiting both during the intraoperative and postoperative phase; (4) the possible appearance of neurological deficits within and after the intervention; (5) the onset of epileptic seizures, which also in this case may be both intra-procedural and post-procedural; (6) the evaluation of the duration of the intervention, the time in which the subjects remained awake (Awake time) and the time in which they remained asleep (Sleep time) and possible correlations of the relationship between them with the quality of the procedure; (7) the degree of patient satisfaction, understood as comfort or state of anxiety, anguish and the presence of pain during the entire operation; (8) moreover, the degree of satisfaction of surgeons, understood as ease of being able to use the procedure during the mapping phase and then evaluation of motor cognitive functions, patient immobility and absence of complications due to failure of anesthesiological management.

## 2. Materials and Methods

### 2.1. Ethical Consideration

The procedure was conducted in accordance with the Declaration of Helsinki and approved by the local Institutional Review Board (Code: ConsInf An/01, approved 6 November 2013). Informed consent given includes approval for the procedure. Further details can be obtained in the protocol, which also includes a description of the protection of data.

### 2.2. Study Sample

From June 2019 to September 2022, we recruited 42 patients undergoing AC in 3 different centers in the south of Italy: Polyclinic “Riuniti” of Foggia, the Research Hospital “Casa Sollievo della Sofferenza” in San Giovanni Rotondo, and the Polyclinic of Bari “Giovanni XXIII”.

The inclusion criteria were the following: (1) presence of brain gliomas (astrocytoma or oligodendroglioma); (2) presence of metastases or arteriovenous malformation (AVM) involving the Broca area; (3) age over 18 years; (4) signed informed consent to the procedure.

### 2.3. Anesthesiological Procedure and Comprehensive Approach

**Figure 1:**
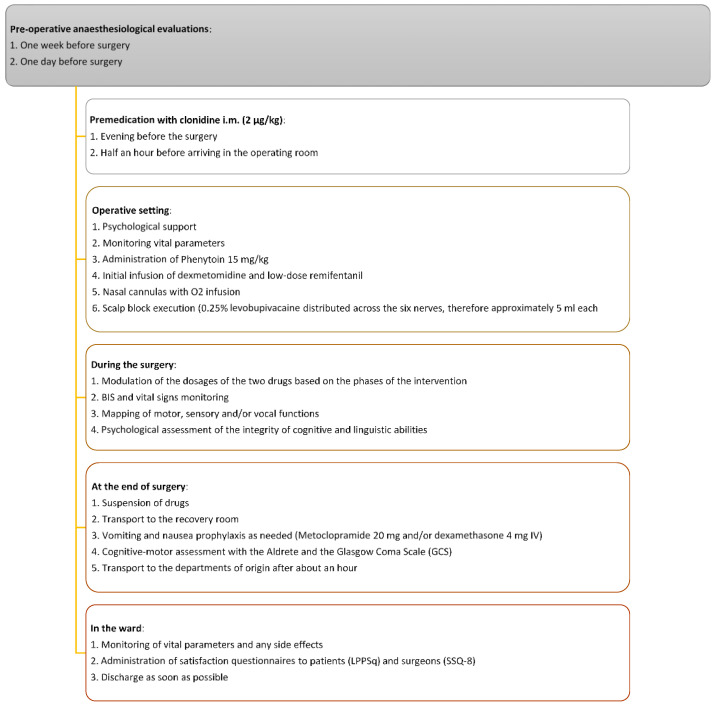
Practical clinical scheme.

As shown in Figure 1, the anesthesiological management is based on a careful preoperative evaluation for each patient at least one week before the procedure and then a re-evaluation on the day of the operation.

Patients were premedicated with intramuscular clonidine (2 µg/kg) the day before surgery and half an hour before arriving in the operating room. The prophylaxis of seizures consists of the administration of Phenytoin 15 mg/kg intravenously before surgery. In the operating room, with the support of the psychologist, patients were placed on the operating table, and their vital signs [oxygen saturation (SpO2), temperature (T), non-invasive blood pressure (nibp), HR, and Spectral Edge Frequency (SEF)] checked, constantly monitored throughout the procedure. At least one large venous access (18–20G), a central venous catheter with an ultrasound-guided technique, and an arterial line for the evaluation of the continuous hemodynamic parameters were found.

**Figure 2:**
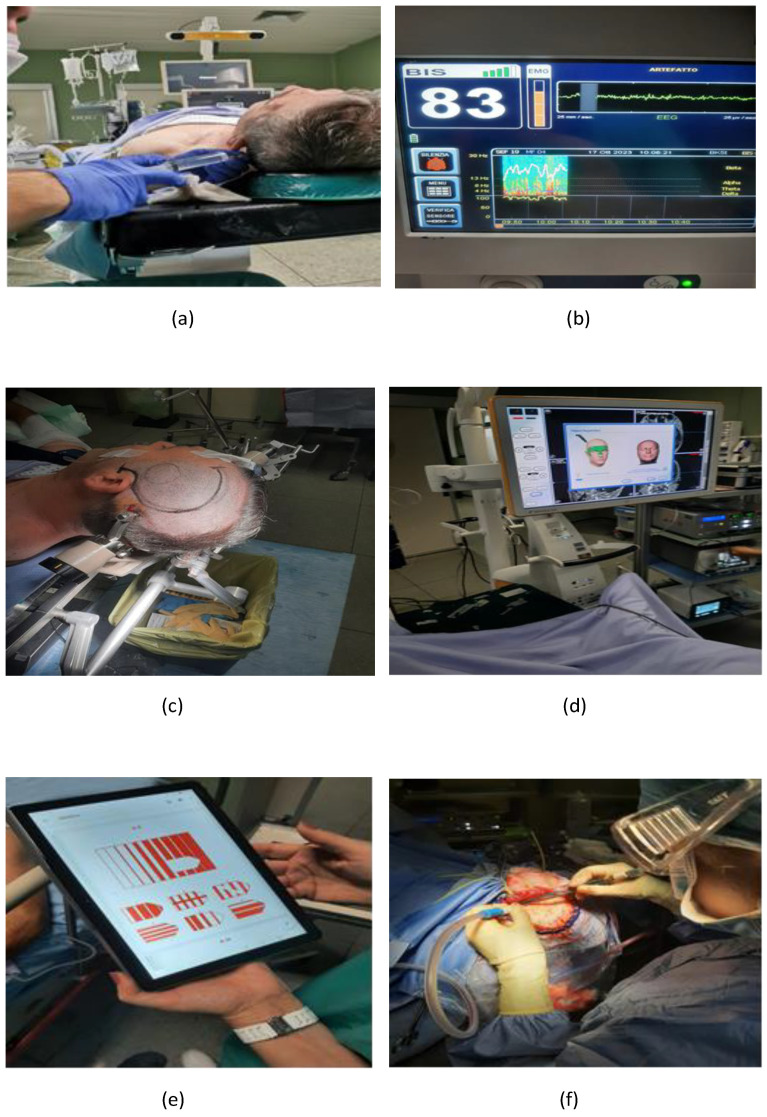
Anesthesiological procedure and comprehensive approach. (**a**) analgesic blockages of the scalp nerves; (**b**) Bispectral analysis (BIS); (**c**) Mayfield’s cranial fixator placed; (**d**) stimulating electrode on the cortical surface; (**e**) Neuropsychological assessment about cognitive and language skill integrity; (**f**) Surgical phase.

The patient followed continuous administration of Dexmedetomidine at low dosages (0.7 µg/kg/h) and Remifentanil via TCI to the site effect with the Minto model, starting with a minimum dosage (0.5–1 ng/mL). Nasal cannulas were placed with O2 infusion to minimize the risk of desaturation and improve tissue oxygenation. All patients breathed spontaneously and received supplemental oxygen at 4 L/min (with an average inhaled 37 fraction of oxygen of about 36%). Afterward, analgesic blockages of the scalp nerves were performed with 0.25% Levobupivacaine without exceeding the maximum allowable dose based on the weight and age of the patients (Figure 2a).

The blocks, in particular, were performed at the level of the six cranial nerves that innervate the scalp bilaterally, distributing the local anesthetic in 30 mL of physiological solution per hemisphere, distributed for the six nerves, then approximately 5 mL each. In case of pain during the surgical procedure, the infusion rates of Dexmedetomidine and/or Remifentanil were increased until the patient responded.

The desired effect to be achieved, to allow the effectiveness of the procedure and the verbal contact, if necessary, with the patient, has been evaluated through the SEF. This value is obtained by bispectral analysis (BIS) and consists of a traditional electroencephalogram (EEG) parameter based on power and is displayed both numerically and graphically (as a white line) in Hertz (Hz) for both cerebral hemispheres (Figure 2b), inside the Density Spectral Array (DSA). The latter being a graphical representation of the power distribution of the EEG frequencies of the patient, the SEF is nothing more than the frequency below which 95% of the power resides on that side of the brain and varies when the strength of the EEG signal moves from one frequency range to the other [9,10]. The effect to be achieved consisted of a range between 16 and 22 Hz, then with the prevalence of β waves in which the patient is adequately comfortable and sleepy but possibly contactable if necessary.

After the patient has been positioned appropriately, the Mayfield’s cranial fixator is placed (Figure 2c). The mapping of motor, sensory, and/or vocal functions takes place by placing a stimulating electrode on the cortical surface (Figure 2d). The anesthesiologist observes every movement of the face, arm, or leg. Motor ability was tested by asking the patient to move his/her hand (fingers) or foot (dorsiflexion) against resistance. Patients were asked to report any changes in sensations.

The role of the psychologist is also useful during this phase as s/he carries out the assessment of the integrity of cognitive and language skills: s/he calls the patient and asks the patient to describe images, pronounce sentences, and/or count numerical series (Figure 2e). This practice is needed to understand if there are any cognitive and/or language alterations when the patient arrived in the operating room and before starting the anesthetic approach, during the preoperative anesthesiological procedure, and during the surgical phase (Figure 2f).

At the end of the surgical procedure, the infusion of the drugs was suspended, and the patient was placed back in the supine position and transported to the recovery room.

Metoclopramide 20 mg and/or Dexamethasone 4 mg intravenous injection (i.i.) were administered for postoperative nausea and vomiting when needed. The Aldrete and Glasgow Coma Scale (GCS) is considered to be the cognitive-motor assessment score at this stage.

Following an observation phase of about 1 h and after a final evaluation of the vital and cognitive parameters, the patients were discharged and transported to the departments of origin. In the ward, they were re-evaluated, monitoring any side effects (pain, headache, nausea, vomiting, dyspnea, seizures, hemodynamic instability, etc.). As soon as possible, the patient was discharged unless there were further complications. At discharge, patient and surgical questionnaires were run. For the patients, the Leiden Perioperative Care Patient Satisfaction Questionnaire (LPPSq) was administered. It is a 39 required 5-point Likert scale response [11].

For the surgeon, the Surgical Satisfaction Questionnaire (SSQ-8), an 8-item questionnaire, were administered. A Likert type scale with responses from 0 “Very Unsatisfied” to 4 “Very Satisfied”, is ranged of scores from 0 to 100 [12].

### 2.4. Statistical Analysis

Out of a population of 60 people with brain gliomas attending the Units of Neurosurgery of the Apulian region (South of Italy) in one year, considering the feasibility of performing surgery in AC (due to the organization of the multidisciplinary operating team and the absence of psycho-behavioral disorders of the patients) only for approximately a quarter of these patients, the estimated minimum number of patients required for three years, assuming a statistical significance at the 5% level and a confidence level of 95%, was n = 41. Finally, 42 patients were recruited for this study.

Demographic and clinical characteristics of preoperative, intra-, and post-operative patients have been reported as averages and standard deviations or frequencies and percentages depending on whether they were continuous variables or categorical variables. The normal distribution of continuous variables was checked using the Shapiro-Wilk test. All analyses were performed using the SPSS software (IBM Corp. Released 2015. IBM SPSS Statistics for Windows, version 23.0. Armonk, NY, USA: IBM Corp). The calculation of the sample size on a number of 42 subjects allows estimating the incidence of the primary endpoint (conversion of the procedure from AC to general anesthesia) with a 95% confidence interval equal to 0.03–11.95 when the actual incidence is 2%.

## 3. Results

### 3.1. Clinical Characteristics of Patients

As shown in Table 1, the population consisted of 27 male (64%) and 15 female (36%) patients with an average age of 58 ± 8 years, and the average weight was 78 ± 11 kg. The resection involved the left temporal area in 21 patients (50%), the left parietal area in 1 patient (2%), the left frontal area in 2 patients (5%), and the left frontal area in 7 patients (17%) the left temporo-parietal area, in 4 patients (9%) the left fronto-parietal area and 7 patients (17%) the left fronto-temporo-parietal area. The type of lesion consisted of 34 patients (81%) with an oligodendroglioma, 4 patients (9%) with an AVM, 2 patients (5%) with an astrocytoma, and 2 patients (5%) with metastases of variable and more or less known origin. The preoperative assessment resulted in preoperative Glasgow coma scale (GCS) scores of 13 in 3 subjects (7%), 14 in 5 subjects (5%), and 15 in 34 subjects (81%). The preoperative risk grade according to the American Society of Anesthesiologists (ASA) classification showed that 2 patients had an ASA1 (5%), 35 patients an ASA2 (83%), and 5 patients an ASA 3 (12%). Only one procedure was interrupted following the onset of an epileptic seizure, promptly treated after placement of the Mayfield clamp and subsequent increase in lactates to the control hemogasanalysis [HGA (5.1 mmol/L)].

### 3.2. Hemodynamic Characteristics

From the point of view of the hemodynamic evaluation, three different moments of the procedure were taken into consideration, i.e., an initial time immediately before surgery, an intermediate time during the evaluation of neurological functions, and a final time at the end of the surgery. Specifically, in Phase 1, average blood pressure (ABP) was around 71 ± 5 mmHg, HR was 76 ± 10 beats per min (bpm), and stroke volume (SV) was 69 ± 13 mL. In Phase 2, a mean ABP of 74 ± 5 mmHg, HR of 79 ± 6 bpm, and SV of 66 ± 14 mL were recorded. Finally, in Phase 3, we obtained an ABP of 72 ± 5 mmHg, HR of 67 ± 9 bpm, and SV of 67 ± 12 mL (Table 2).

### 3.3. Respiratory Characteristics

Regarding the respiratory component (Table 3), the three phases were taken into account in the same way, and the SpO2 was evaluated; furthermore, an arterial blood sample was carried out for blood gas analysis, from which pulmonary hypertension (PH) data, partial arterial pressure of O2 (PaO2), partial arterial pressure of CO2 (PaCO2), the ratio of PaO2 to the inspiratory fraction (P/F) of O2, which we recall was about 36%, the bicarbonate (HCO3−) value and the lactate level were found. An average SpO2 (in %) of 98 ± 1 was recorded in Phase 1, 99 ± 1 in Phase 2, and a SpO2 of 98 ± 1 in Phase 3.

From the HGA carried out in Phase 1, an average PH of 7.41 ± 0.04, an average PaO2 of 115 ± 22, an average P/F of 326 ± 61, an average PaCO2 of 40 ± 4, a HCO3—average of 24 ± 2 and lactates of 0.9 ± 0.3. In the second phase, mean HGA values of PH of 7.4 ± 0.04, of PaO2 of 122 ± 18, of P/F of 366 ± 51, of PaCO2 of 40 ± 2, of HCO3− of 24 ± 2 were recorded, and of lactates of 1 ± 0.3. Finally, in the third phase, at the HGA, an average PH of 7.4 ± 0.05, a PaO2 of 120 ± 20 mmHg, an average PaCO2 of 35 ± 3 mmHg, an average P/F of 325 ± 47, a HCO3− of 23 ± 2 mmol and lactate levels of 1 ± 0.7 mmol/L were found.

### 3.4. Side Effect Detected

There were no intraoperative side effects (seizures, aphasia, dyspnea, agitation, panic attacks) for which it was necessary to suspend the procedure except for one patient (2%) in which it was necessary to interrupt the operation before the incision of the epileptic seizure skin. In two patients (5%), agitation was observed due to anxiety and reported annoyance in the incision area, for which the surgery was immediately stopped, and the dosage of drugs for analgo-sedation in continuous infusion increased; it was possible to complete the surgical procedure without further problems. In 3 patients (7%), a clinically tested intraoperative aphasia occurred, which was fortunately transient and resolved spontaneously, without the need to interrupt the procedure and/or modify the anesthesiological conduct (Table 4).

### 3.5. Procedural Steps

As shown in Table 5, the total duration of the interventions was, on average, 240 ± 62 min; in this period of time, it is necessary to consider the time in which the patient remained asleep (Sleep time) and the time in minutes in which the patient remained completely awake (Awake time). In general, the mean sleep time (ST) was 120 ± 45 min, while the mean awake time (AT) was 120 ± 32 min. The desired effect, i.e., a physiological sleep in which the patient maintains spontaneous breathing and remains in a state of comfort but as needed, reducing the dosage of drugs, awakening, and collaborating for evaluation and mapping, was evaluated not only clinically but also through the observation of the pulsed electrical stimulation (PES) that in these cases was necessary to maintain at a frequency range between 16 and 20 Hz; the average SEF evaluated to achieve the desired effect was 18 ± 1 Hz. The average dosages of Dexmedetomidine to achieve this value were 0.7–1.2 µ/kg/h, while Remifentanil doses were 0.08–0.1 µ/kg/h. The Time to Peak Emptying (TtPE) to achieve this aim averaged 142 ± 8 s. From the moment of the reduction of the dosage of the drugs in continuous infusion to the total awakening of the patient and, therefore, to the achievement of a significant increase in the PES (24–35 Hz), the tD was calculated obtaining an average time of 94 ± 7 s. The total amount of Remifentanil and Dexmedetomidine infused during the interventions was calculated. Regarding Remifentanil, we obtained an average quantity of 4.2 ± 1.3 mg of the drug, while for Dexmedetomidine, a total of 1.0 ± 0.3 mg on average.

### 3.6. Postoperative Evaluation

No significant side effects were described in the post-operative phase, especially during observation in the recovery room and after transfer to the wards of origin. As shown in Table 6, postoperative GCS assessed at 1 h postoperatively was 12 in 1 patient (2%), 13 in 2 patients (5%), 14 in 2 patients (5%), and 15 in 37 patients (88%). For the Aldrete scale, a score of 10 was observed in 38 patients (91%), a score of 8 in 3 patients (7%), and a score of 7 in 1 patient (2%).

### 3.7. Patient and Surgeon Satisfaction Evaluation

**Figure 3:**
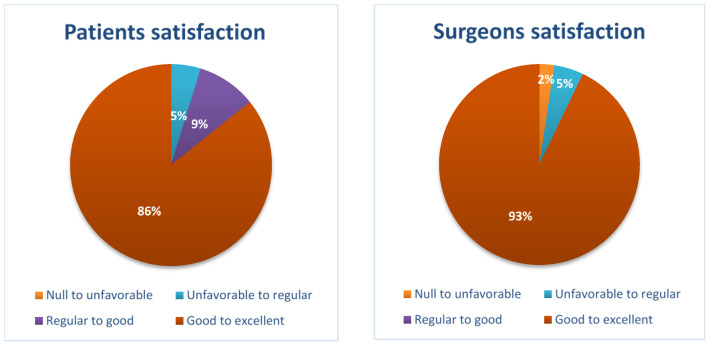
Patient and surgeon satisfaction, assessed by Leiden Perioperative care Patient Satisfaction questionnaire (LPPSq) and Surgical Satisfaction Questionnaire (SSQ-8).

After the operation, the patient was asked to rate his/her satisfaction with the entire preoperative anesthetic management, considering a score of 1 in case s/he felt totally unsatisfied, a score of 2 in cases when s/he felt partially satisfied, 3 almost completely satisfied and 4 totally satisfied. In this perspective, 2 patients expressed a score of 2 (5%), 4 patients a score of 3 (9%), and 36 patients a score of 4 (86%). The same type of question was asked to the neurosurgeons operating for a general approval of the intervention. These responded by giving a score of 1 (2%) in only one case, i.e., the patient who unfortunately was not operated on in that circumstance due to an epileptic seizure, a score of 2 in 2 cases (5%), and a score of 4 in the remaining 39 cases (93%) (Figure 3).

## 4. Discussion

Regarding the primary endpoint, no conditions, such as causing the interruption of the procedure or converting the anesthetic management to general anesthesia, were recorded, except in one case. This result is consistent with the data registered in most studies, where it was reported that AC failure, defined as the inability to complete intraoperative pacing mapping or the need to convert to general anesthesia, is rare and occurs in less than 2% of cases regardless of the anesthetic technique used. The literature confirms what was assessed in our study: there is rarely a need for emergency airway management, although the full range of airway equipment, including a laryngeal mask, video laryngoscope, and endotracheal tube, should be readily available throughout the procedure [13]. We also recorded a high level of hemodynamic stability in most of the recruited patients. A small number of intra- and post-procedural adverse effects were observed in the study.

Intraoperative monitoring is essential as it helps prevent any clinical events of instability; in particular, the monitoring of cerebral electrical activity through the evaluation of the PES thanks to the DSA extrapolated from the EEG is important to better understand the sedation plan and possibly modify the infusion rates of the drugs [14,15].

The efficacy of intraoperative seizure prophylaxis with anticonvulsants remains questionable. No benefits of prophylaxis have emerged since the last systematic review on this issue [16]. The possible triggering of convulsions is a very serious factor that could invalidate the procedure and cause major problems for the patient [17]. However, it has been highlighted in the literature that the risk of triggering a seizure during the awakened craniotomy is very low. The main risk factor is a history of preoperative seizures and anterior tumor location. Prophylaxis with an anticonvulsant may not be necessary, even if there are no precise recommendations in this regard [18]. It should be noted, however, that most of the evidence on seizure prevention is based on the use of phenytoin or valproate. On the other hand, there is new data supporting the superiority of levetiracetam in this context but not sufficiently to recommend its routine use [16]. The main risk factor is a history of preoperative seizures. Electrocorticographic monitoring of epileptic activity intraoperatively and irrigation of brain tissue with ice-cold crystalloid solution could stop a seizure or prevent it from generalizing in a timely manner. In case of ineffective irrigation, administering low doses of propofol may be recommended [18]. The position of the patient is important since the stress of the operation can be worsened by a condition of pain due to an incorrect posture maintained for long periods of time [19]. This entails a lesser predisposition to collaborate, to remain motionless during resections of the areas at risk of permanent lesion, and above all, a greater need for analgesic drugs and, therefore, a greater risk of adverse events or respiratory depression [20]. Typically, the cause of a permanent deficit is an ischemic lesion of the subcortical structures (internal capsule, etc.) or anatomical damage of the subcortical pathways. These complications are often associated with errors in mapping methodology, excessive sedation, or awake patient fatigue. It appears that the most effective way to avoid these complications is to use a transcortical approach to deep structures, which prevents vascular traction and reduces the risk of ischemic damage. Furthermore, such an approach can prevent fatigue and pain because the sensitive tissue around the vessels and leptomeninges remains intact [21]. Good knowledge of the location of the lesion and adequate intraoperative mapping further reduce the risks derived from an accidental injury in unaffected areas [22]. Furthermore, the experience of the surgeons naturally reduces the risks related to excessive hemorrhagic losses and, therefore, significant cardiovascular alterations, conditions linked to seizure triggers, and altered epileptogenic foci, which necessarily involve the use of drugs, which can modify the anesthesiological plan [23].

The support of the psychologist in this type of patient, who already has a dramatic familial and social experience and condition, is all the more important in a condition in which these subjects must undergo an intervention of such magnitude that can at any time involve irreversible risks, in a state of total or near total consciousness [24]. This is why the presence of a professional who can minimize both pre- and intra-procedure stress and discomfort is essential, if not impossible, to eliminate [3].

Among the results obtained, those that seemed most significant to us for an assessment of the quality of the type of anesthesia used and the comfort of the patient are the following: Sleep time and Awake time are, on average, superimposable, which is an indication of the fact that once the patient is superficial, very often at the end of the evaluation of the functions of the language, it was no longer necessary to study it again [25]. This may indicate that the patient, feeling comfortable, was not required to be put back to sleep. This condition was confirmed by the monitored vital parameters.

The relationship between the total dosages of dexmedetomidine and remifentanil presented a high variability between patients regardless of the duration of the intervention. The significance of this result confirms that it is not possible to consider standard dosages with which to make uniform evaluations. This concept allows us to understand why it is important to follow the phases of the intervention and anticipate the needs of the patient and the surgeon, modifying the dosages of the two drugs also on the basis of the different TTPE and DT of the substances. The latter was found to be quite adequate for the operative needs; in particular, obtaining an average DT of less than 2 min indicates that the speed and infused quantity of Dexmedetomidine was adequately calculated, which otherwise, due to its pharmacokinetic characteristics that have been discussed, would cause accumulation and difficulty in early recovery, reduced quality of intraoperative assessment, and delays in procedures.

To reinforce the validity of this concept, another result was obtained, namely the approval of the surgeons, who gave a maximum score in 93% of cases. Finally, as far as patient satisfaction is concerned, which for us was probably the most important result, we obtained a maximum score for 86% of patients here too.

To conclude, we would first like to underline how much work it takes to be able to formulate a valid and complete protocol in the peri-operative management of a patient subjected to AC. This requires a well-oiled and trained work sector to best face any adverse conditions, possibly modifying the previously agreed conduct and opting for the best one that can solve the current problem. The coordination of the team and the preoperative preparation of the management of each patient are the keys to obtaining the best result both from an anesthesiologic and surgical point of view [26]. It must be considered that it is not easy for a patient who accepts to undergo this type of procedure while remaining awake [27]. It is sufficient to say that the first request that any type of patient normally makes is to sleep, not to see or feel anything, and to wake up only after the fact. Appropriate patient selection and excellent multidisciplinary teamwork are associated with high levels of procedural success and patient satisfaction [19].

## 5. Conclusions

Although the optimal anesthetic schedule remains a debatable concept, it is clear that a reasonable combination of modern anesthetics and comprehensive psychological support in the perioperative period can ensure successful intraoperative brain mapping in nearly all cases. The correct execution of scalp nerve blocks is necessary to obtain a valid initiation of surgery without having to convert the anesthetic management. The synergy between opioid drugs and α2 agonists plays a fundamental role in ensuring the success of the procedure. Stronger evidence could be provided by randomized controlled trials [6].

## Figures and Tables

**Table 1:** Description of the population.

	Patients (n = 42)
**Sex** (Male/Female)—n(%)	27(64.3%)/15(35.7%)
**Age**—(mean ± sd)	58 ± 8
**Weight**—(mean ± sd)	78 ± 11
**Surgery area**	
Temporal sn—n(%)	21(50%)
Parietal sn—n(%)	1(2%)
Frontal sn—n(%)	2(5%)
Temporo-parietal sn—n(%)	7(17%)
Fronto-parietal sn—n(%)	4(9%)
Fronto-temporo-parietal sn—n(%)	7(17%)
**Type of lesion**	
Oligodendroglioma—n(%)	34(81%)
AVM—n(%)	4(9%)
Astrocitoma—n(%)	2(5%)
Metastasi—n(%)	2(5%)
**ASA**	
1—n(%)	2(5%)
2—n(%)	35(83%)
3—n(%)	5(12%)
4—n(%)	0(0)
**GCS preoperative**	
13—n(%)	3(7%)
14—n(%)	5(12%)
15—n(%)	34(81%)
**Suspension of the procedure—n(%)**	1(2%)

Legend: AVM, arteriovenous malformation; ASA, American Society of Anesthesiologists; GCS, Glasgow Coma Scale.

**Table 2:** Hemodynamic parameters.

Phase	Parameter	Patients (n = 42)
Phase 1	**ABP** (mmHg)—(mean ± sd)	71 ± 5
	**HR** (bpm)—(mean ± sd)	76 ± 10
	**SV** (mL)—(mean ± sd)	69 ± 13
Phase 2	**ABP** (mmHg)—(mean ± sd)	74 ± 5
	**HR** (bpm)—(mean ± sd)	79 ± 6
	**SV** (mL)—(mean ± sd)	66 ± 14
Phase 3	**ABP** (mmHg)—(mean ± sd)	72 ± 5
	**HR** (bpm)—(mean ± sd)	67 ± 9
	**SV** (mL)—(mean ± sd)	67 ± 12

Legend: ABP, average blood pressure; HR, heart rate; SV, stroke volume.

**Table 3:** Respiratory parameters.

Phase.	Parameter	Patients (n = 42)
Phase 1	**SpO2** (%)—(mean ± sd)	98 ± 1
	**PH**—(mean ± sd)	7.41 ± 0.04
	**PaO2** (mmHg)—(mean ± sd)	115 ± 22
	**P/F**—(mean ± sd)	326 ± 61
	**PaCO2** (mmHg)—(mean ± sd)	40 ± 4
	**HCO3**− (mmol/L)—(mean ± sd)	24 ± 2
	**Lactate** (mg/dl)—(mean ± sd)	0.9 ± 0.3
Phase 2	**SpO2** (%)—(mean ± sd)	99 ± 1
	**PH**—(mean ± sd)	7.40 ± 0.04
	**PaO2** (mmHg)—(mean ± sd)	122 ± 18
	**P/F**—(mean ± sd)	336 ± 51
	**PaCO2** (mmHg)—(mean ± sd)	40 ± 2
	**HCO3**− (mmol/L)—(mean ± sd)	24 ± 2
	**Lactate** (mg/dL)—(mean ± sd)	1.0 ± 0.3
Phase 3	**SpO2** (%)—(mean ± sd)	98 ± 1
	**PH**—(mean ± sd)	7.40 ± 0.05
	**PaO2** (mmHg)—(mean ± sd)	120 ± 20
	**P/F**—(mean ± sd)	325 ± 47
	**PaCO2** (mmHg)—(mean ± sd)	35 ± 3
	**HCO3**− (mmol/L)—(mean ± sd)	23 ± 2
	**Lactate** (mg/dL)—(mean ± sd)	1.0 ± 0.7

Legend: SpO2, oxygen saturation; PH, pulmonary hypertension; PaO2, partial arterial pressure of O2; P/F, PaO2 to the inspiratory fraction; PaCO2, arterial partial pressure of carbon dioxide; HCO3−, bicarbonate.

**Table 4:** Side effects.

Effect	Patients (n = 42)
**Epilepsy**—n(%)	1(2%)
**Agitation**—n(%)	2(5%)
**Aphasia**—n(%)	3(7%)

**Table 5:** Procedural times, Spectral Edge Frequency, and Dexmedetomidine and Remifentanil doses.

Feature	Patients (n = 42)
**Duration of procedure** (min)—(mean ± sd)	240 ± 62
**Awake time** (min)—(mean ± sd)	120 ± 45
**Sleep time** (min)—(mean ± sd)	120 ± 32
**Ttpe** (s)—(mean ± sd)	142 ± 8
**tD** (s)—(mean ± sd)	94 ± 7
**Dose of Remifentanil** (mg)—(mean ± sd)	4.2 ± 1.3
**Dose of** Dexmedetomidine (µg)—(mean ± sd)	996 ± 316
**SEF** (Hz)—(mean ± sd)	18 ± 1

Legend: Ttpe, Time to Peak Emptying; tD, time-domain; SEF, Spectral Edge Frequency.

**Table 6:** Postoperative evaluation.

	Patiens (n = 42)
**Aldrete scale**	
10—n(%)	38(91%)
8—n(%)	3(7%)
7—n(%)	1(2%)
**Postoperative GCS**	
12—n(%)	1(2%)
13—n(%)	2(5%)
14—n(%)	2(5%)
15—n(%)	37(88%)

Legend: GCS, Glasgow Coma Scale.

## Data Availability

The data presented in this study are available on request from the corresponding author. The data is not publicly available due to patient privacy concerns.
